# Application of Machine Learning Techniques to Assess Alpha-Fetoprotein at Diagnosis of Hepatocellular Carcinoma

**DOI:** 10.3390/ijms25041996

**Published:** 2024-02-07

**Authors:** Sergio Gil-Rojas, Miguel Suárez, Pablo Martínez-Blanco, Ana M. Torres, Natalia Martínez-García, Pilar Blasco, Miguel Torralba, Jorge Mateo

**Affiliations:** 1 Gastroenterology Department, Virgen de la Luz Hospital, 16002 Cuenca, Spain; 2 Medical Analysis Expert Group, Institute of Technology, Universidad de Castilla-La Mancha, 16071 Cuenca, Spain; 3 Medical Analysis Expert Group, Instituto de Investigación Sanitaria de Castilla-La Mancha (IDISCAM), 45071 Toledo, Spain; 4 Internal Medicine Unit, University Hospital of Guadalajara, 19002 Guadalajara, Spain; 5 Department of Pharmacy, General University Hospital, 46014 Valencia, Spain; 6 Faculty of Medicine, Universidad de Alcalá de Henares, 28801 Alcalá de Henares, Spain; 7 Translational Research Group in Cellular Immunology (GITIC), Instituto de Investigación Sanitaria de Castilla-La Mancha (IDISCAM), 45071 Toledo, Spain

**Keywords:** hepatocellular carcinoma, liver cirrhosis, alpha-fetoprotein, machine learning, mortality, prognosis

## Abstract

Hepatocellular carcinoma (HCC) is the most common primary liver tumor and is associated with high mortality rates. Approximately 80% of cases occur in cirrhotic livers, posing a significant challenge for appropriate therapeutic management. Adequate screening programs in high-risk groups are essential for early-stage detection. The extent of extrahepatic tumor spread and hepatic functional reserve are recognized as two of the most influential prognostic factors. In this retrospective multicenter study, we utilized machine learning (ML) methods to analyze predictors of mortality at the time of diagnosis in a total of 208 patients. The eXtreme gradient boosting (XGB) method achieved the highest values in identifying key prognostic factors for HCC at diagnosis. The etiology of HCC was found to be the variable most strongly associated with a poorer prognosis. The widely used Barcelona Clinic Liver Cancer (BCLC) classification in our setting demonstrated superiority over the TNM classification. Although alpha-fetoprotein (AFP) remains the most commonly used biological marker, elevated levels did not correlate with reduced survival. Our findings suggest the need to explore new prognostic biomarkers for individualized management of these patients.

## 1. Introduction

Hepatocellular carcinoma (HCC) is the seventh most frequently diagnosed cancer worldwide, with approximately 906,000 new cases reported in 2020 [1]. With a five-year survival rate of approximately 18%, it represents the third most common cause of cancer-related mortality worldwide [2,3]. Incidence and mortality rates have increased in many parts of the world, primarily due to the large number of undiagnosed chronic hepatitis C virus (HCV) infections, rising alcohol consumption, and metabolic dysfunction-associated steatotic liver disease (MASLD) [4,5]. The epidemiology of HCC is changing. This is based on hepatitis B vaccination policies and new hepatitis C treatments, which have led to a decrease in secondary cases of these diseases. Despite this good news, the increase in the incidence of patients with MASLD contributes to the sustained high figures of this tumor’s impact on cancer-related deaths [6].

Most of these tumors occur in patients with liver cirrhosis [7]. As most patients are diagnosed in this situation, they often present a deteriorated general condition as a result. Without considering common situations such as malnutrition or other comorbidities, patients will exhibit altered liver function [8]. This also implies that, in the face of any hepatic decompensation, it is necessary to rule out the presence of HCC in these patients [7]. There are various prognostic and therapeutic classifications of HCC, and none of them is universally valid. This is because different geographic areas have distinct risk factors that can modify the course and prognosis of the disease [9,10]. Furthermore, no molecule capable of predicting the course of these tumors has been identified or included in staging systems. The Barcelona Clinic Liver Cancer (BCLC) classification is widely used in our context, proving particularly useful for approximately 70% of patients not eligible for curative intent treatment at the time of diagnosis [11]. The implementation of appropriate screening programs for early-stage detection is of great importance. It is recommended to perform a surveillance ultrasound every 6 months in all cirrhotic patients, apart from those in Child–Pugh stage C who are not candidates for liver transplantation, non-cirrhotic patients with hepatitis B virus (HBV) infection, and those with advanced fibrosis (F3–F4). The determination of alpha-fetoprotein (AFP) levels is not highly sensitive or specific for screening, and its use does not seem justified [12].

The poor prognosis of this disease demands further research. It is essential to identify parameters and biomarkers that enable earlier detection of HCC and establish a prognosis upon diagnosis. This prognosis is crucial for determining the most optimal treatments in each case and providing better information regarding survival. Current evidence regarding biomarkers is limited and inadequate [13]. There are multiple avenues under investigation, many of which are focused on liquid biopsy, the presence of circulating tumor DNA and cells, microRNA in blood, and metabolomics, among others [14,15]. All of these are far from validation and application in routine clinical practice. Therefore, currently, in many cases, AFP levels are used due to their traditional application, accessibility, and being one of the few available options [16].

If the use of AFP in screening is controversial, its utility as a prognostic factor is also a subject of debate. Despite its classical use, there are doubts about its effectiveness as a prognostic factor at the time of HCC diagnosis [12,17]. It has been suggested that elevated AFP levels may have prognostic implications, as an inversely proportional relationship with patient survival has been observed [7]. Moreover, a significant elevation of AFP levels above 1000 mcg/L has been associated with an increased risk of recurrence in transplanted patients, regardless of tumor size [18]. Given the limited evidence regarding the use of AFP to predict prognosis in these tumors, the following study is proposed to analyze whether a real relationship exists between AFP levels and patient survival. For this purpose, machine learning (ML) techniques have been implemented. Compared to conventional statistics, ML employs mathematical algorithms that can analyze many variables and uncover patterns that are not accessible through these statistical techniques [19,20]. The eXtreme gradient boosting (XGB) method has been proposed as the algorithm for the development of the model. This algorithm has been selected for its characteristics in terms of execution speed, scalability, and the utilization of regularization techniques [21]. This method has already been utilized in other medical fields, including hepatology. Through the application of this algorithm, the aim is to develop a predictive model to assess the utility of AFP as a prognostic factor for mortality in the diagnosis of HCC.

## 2. Results

In the retrospective cohort obtained from the coding records of two hospital centers in Castilla-La Mancha (Cuenca and Guadalajara), a total of 208 patients diagnosed with HCC, both through histological and radiological studies, were identified between the years 2008 and 2022.

**Figure 1:**
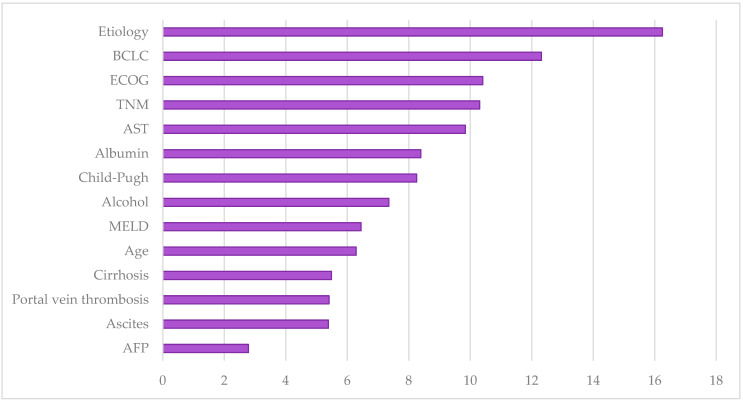
Representation of the weight of each variable within the machine learning predictive model. BCLC: Barcelona Clinic Liver Cancer, ECOG: Eastern Cooperative Oncology Group, TNM: tumor nodes metastases, AST: aspartate aminotransferase, MELD: model for end-stage liver disease.

Figure 1 depicts the importance of the variables in constructing the predictive model using ML methods. The etiology of HCC was the most crucial variable, followed by the BCLC classification, which proved superior to TNM. Alcohol consumption was the etiological factor associated with a worse prognosis, emerging as an independent predictive factor for mortality in the study. The Eastern Cooperative Oncology Group (ECOG) general status scale and the Child–Pugh scale, all included in the BCLC classification, also emerged as independent predictive factors for mortality. Other prognostic factors included levels of aspartate aminotransferase (AST), albumin, and the presence of ascites, encompassed in hepatic functional reserve classifications such as Child–Pugh and MELD. However, AFP turned out to be a less significant variable, highlighting the lack of correlation between its isolated elevated levels and the prognosis of these patients.

Table 1 and Table 2 present the results of the different ML methods used: Support vector machine (SVM), Bayesian linear discriminant analysis (BLDA), decision tree (DT), Gaussian naïve Bayes (GNB), K-nearest neighbors (KNN), and the proposed XGB system. As observed, GNB achieved the lowest accuracy, barely surpassing 80%; BLDA and DT obtained slightly higher values, though not reaching 85%; and SVM showed higher accuracy with values exceeding 86%. The proposed XGB system achieved accuracy values surpassing 95%, representing a difference of over 6% compared to the second-ranking KNN method. This translates to a significant improvement in prediction compared to the other proposed algorithms. As seen in Table 1 and Table 2, the same trend occurs when analyzing precision, recall, and F1 score for the different proposed methods. XGB obtained higher values compared to other algorithms, indicating better classification of the study variables.

To assess the performance of the proposed XGB method, other commonly used parameters in the scientific literature were employed. These included area under the curve (AUC), kappa index, Matthews correlation coefficient (MCC), and degenerate Youden index (DYI). For this analysis, MCC serves as the best parameter to discern whether the prediction has been accurately made across the four categories of the confusion matrix (true positives, false positives, true negatives, and false negatives). The results in the four categories of the matrix are proportional to the size of positive and negative elements in the dataset. As can be observed in Table 1 and Table 2, the proposed XGB method achieved a value of 84.46%, clearly superior to the values obtained by the other algorithms. The second-ranking algorithm in classification is KNN, with a value of 79.28%, followed by SVM with 76.95%. Regarding the kappa index, XGB obtained a value of 84.74%, surpassing the values of KNN and SVM by 5.2% and 7.53%, respectively. The same trend is observed when analyzing AUC and DYI, where XGB achieved the highest values, indicating a better prediction of prognostic factors at the diagnosis of HCC.

**Figure 2:**
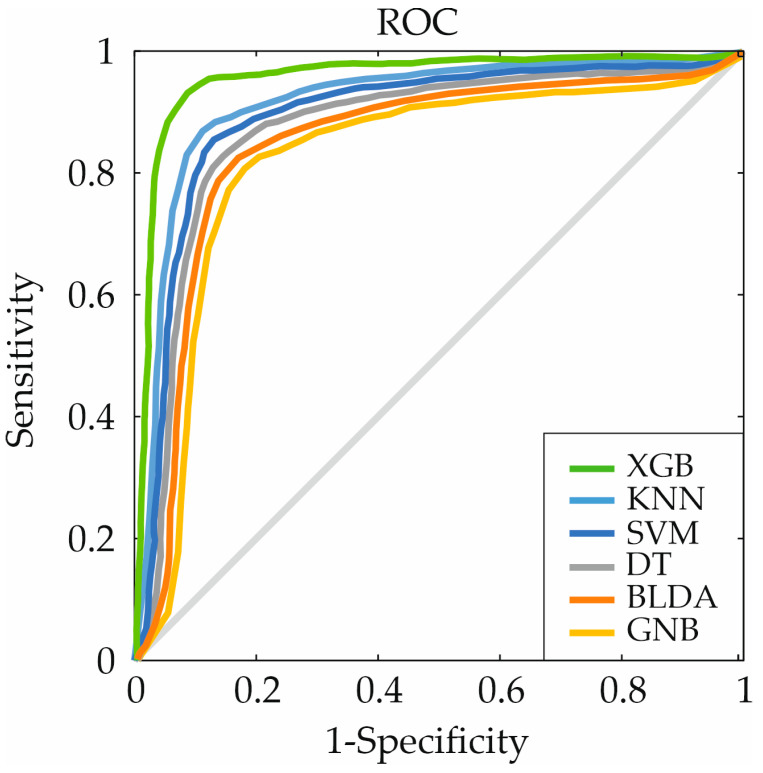
ROC curves for the six machine learning algorithms. ROC: receiver operating characteristic, XGB: eXtreme gradient boosting, KNN: K-nearest neighbors, DT: decision tree, SVM: support vector machine, BLDA: Bayesian linear discriminant analysis, GNB: Gaussian naïve Bayes.

On the other hand, Figure 2 represents the receiver operating characteristic (ROC) curve comparing the XGB system with the other algorithms used. The curve is plotted based on sensitivity and specificity for each threshold value. As can be seen in Figure 2, the XGB method obtains a larger area under the curve, indicating it as the best algorithm for the study’s objective. The various AUC values can be referred to in Table 1. XGB achieves an AUC value of 0.95, followed by KNN with a value of 0.89.

**Figure 3:**
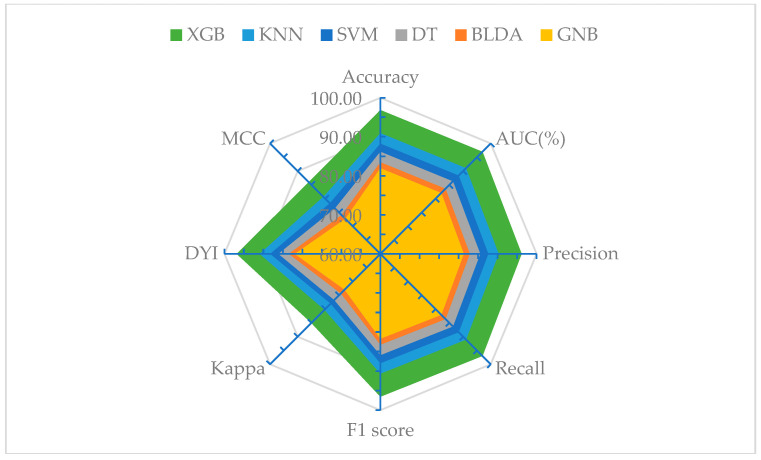

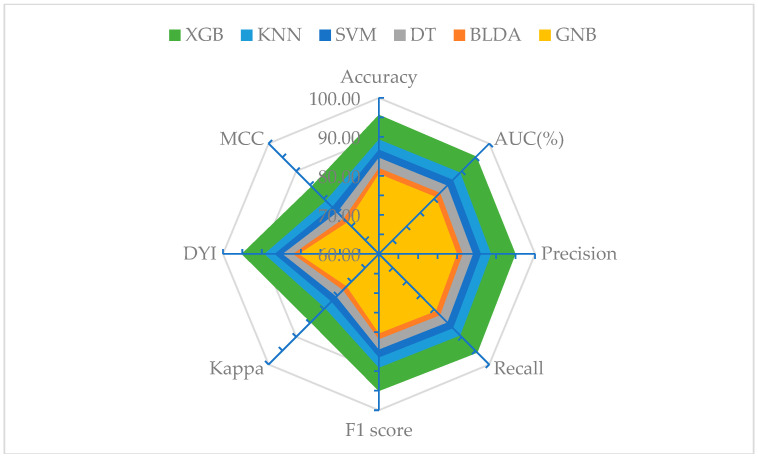
Radar plot of the training phase (**above**) and validation (**below**) to establish the importance of the different prognostic factors in hepatocellular carcinoma within the predictive model. SVM: support vector machine, BLDA: Bayesian linear discriminant analysis, DT: decision tree, GNB: Gaussian naïve Bayes, KNN: K-nearest neighbors, XGB: eXtreme gradient boosting.

To synthesize all the parameters analyzed across different algorithms, a radar plot has been compiled. This representation includes all metrics and displays them as a circle of the entire grid size in Figure 3. The larger the area of the circle, the better the predictive method. The performances of various ML methods validated in the scientific community were compared. The proposed XGB system was able to classify patients in line with the study’s purpose with high precision, proving to be a balanced method. The similarity obtained between the training and validation phases of the test explains the absence of overfitting in this method, making it highly generalizable. Furthermore, XGB is characterized by automatically classifying patients, making it a valuable tool in clinical practice. As shown in Figure 3, the GNB algorithm yielded the worst results for all parameters.

## 3. Discussion

**Figure 4:**
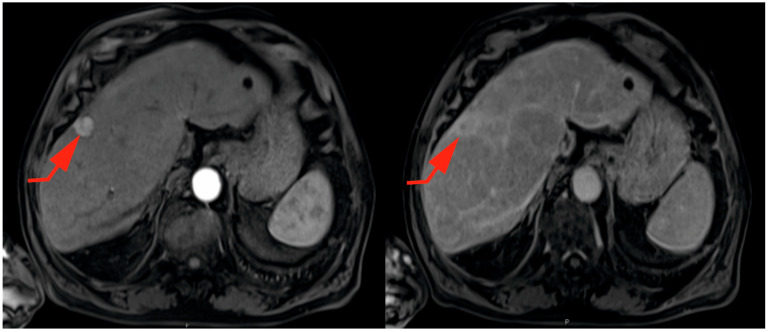
Axial image depicting the typical behavior of HCC on contrast-enhanced liver magnetic resonance imaging (MRI). It shows a hyperenhancing subcapsular focal lesion in the arterial phase (left), with isoattenuation and washout in the portal phase (right). The red arrow indicates the lesion in both phases. This is observed in a patient exhibiting signs of chronic liver disease. Additionally, a cyst is evident in segment II of the left hepatic lobe.

Up to one-third of patients with cirrhosis will develop HCC during their lifetime, with an annual incidence rate ranging from 1 to 7%, as reported in long-term follow-up studies [7]. Chronic infection with HBV and HCV is responsible for more than 50% of diagnosed cases worldwide. Patients with chronic HBV infection are at risk of developing HCC, even in the absence of cirrhosis [5]. Universal vaccination of newborns against HBV and the development of antiviral treatments for HCV contribute to a decrease in the risk of developing HCC [22,23]. Other factors associated with the development of these tumors include hemochromatosis, Wilson’s disease, primary biliary cirrhosis (PBC), autoimmune hepatitis (AIH), alpha-1 antitrypsin deficiency, and environmental aflatoxins [2,24]. This tumor typically appears around the sixth decade of life and has 2 to 3 times higher incidence and mortality rates in men [12]. Spain is considered a region with an intermediate incidence of primary liver cancer, with approximately 4.8–6 cases per 100,000 inhabitants per year. In the conducted study, the cumulative incidence was 3 cases of HCC per 100,000 inhabitants per year for a population of close to 462,000 residents across the two hospital centers. Considering that HCC is the most common primary liver tumor, following intrahepatic cholangiocarcinoma and mixed differentiation hepatic tumors, in our analyzed geographical area, the incidence of HCC was somewhat lower compared to the rest of the country [7,12]. The difference can be explained by the management of this tumor in each hospital. There are cases diagnosed in other medical services for which the necessary data were not available for this study. Its diagnosis is often delayed due to the absence of early symptoms and the presence of very nonspecific symptoms related to chronic liver disease [22,25]. Although there are continuous advances in imaging techniques that have modified diagnostic criteria in cirrhotic patients and new therapeutic options are being developed, the fact that these patients often start with poor functional liver reserve limits the possibility of receiving curative treatment [26,27]. Since these tumors exhibit exclusively arterial vascularization, unlike the hepatic parenchyma, which has a mixed vascular supply (portal and arterial), typical radiological images of their behavior can be obtained through dynamic imaging tests such as computed tomography (CT) or magnetic resonance imaging (MRI), as can be observed in Figure 4 [7]. This characteristic image is a result of histological changes secondary to the presence of hepatic cirrhosis. The fibrous and inflammatory tissue that replaces the normal architecture of the liver in these patients hinders the flow of blood through the portal vein. Increased vascular resistance promotes the development of portal hypertension, contributing to the creation of a hypoxic environment. In order to survive this unfavorable environment, the formation of new blood vessels takes place, enhancing the arrival of blood from the hepatic artery [28,29]. Due to the occurrence of a neoangiogenic process during tumor development, replacing venous vascularization with a purely arterial one, imaging techniques have been developed to enable diagnosis in cirrhotic livers without the need for a liver biopsy. However, histological confirmation is necessary in non-cirrhotic patients or those with liver cirrhosis who do not present a typical radiological pattern according to LI-RADS criteria in dynamic imaging tests [30]. The enormous heterogeneity in this type of tumor makes it relevant to have other prognostic factors that can improve the survival of these patients [17,31].

AFP is a glycoprotein produced in the yolk sac, fetal liver, and gastrointestinal tract during gestation. The AFP gene is located on chromosome 4 in the region 4q11-q13 [32]. It is a 69 to 70 kDa protein that belongs to the albuminoid gene family, along with albumin, vitamin D-binding protein, and alpha-albumin [33]. The concentration of this protein in fetal serum increases until the second trimester of pregnancy, reaching peaks of 3 mg/mL. Its levels in adults are extremely low compared to albumin synthesis [34,35]. Elevated levels of AFP in adults can be found in HCC and other tumor diseases (germ cell tumors, cholangiocarcinoma, and gastric adenocarcinoma). They can also be elevated in benign liver diseases and in processes of liver regeneration (viral hepatitis or drug-induced hepatitis, and cirrhosis) [36,37].

Although AFP continues to be the most widely used serum biomarker in the diagnosis of patients with HCC, its role remains controversial [38]. There are different recommendations in various international clinical practice guidelines due to their low sensitivity and specificity and the lack of established cutoff values [39,40]. American and European clinical practice guidelines do not recommend its determination as a useful screening tool because up to 80% of small-sized HCCs (<2 cm) do not show elevated values [9,12]. However, Asian clinical guidelines and some recent meta-analyses, such as the one conducted by Colli et al., suggest its determination in screening programs for these patients, combined with abdominal ultrasound every 6 months [26,41]. Different AFP levels have been described in relation to the etiology of HCC and the histological subtype, making their determination in screening programs especially useful in countries with a high incidence of HCC [42]. The recent study led by Oh et al. demonstrated the clinical significance of AFP determination in HCC screening in an endemic area. According to this study, frequent AFP determination was independently associated with an overall increase in patient survival, facilitating early-stage detection and the likelihood of receiving curative treatments upon diagnosis. Among the various etiologies of HCC, patients with HBV infection obtained greater benefits in relation to an increased frequency of AFP determinations [43].

Different studies that have analyzed AFP levels as a prognostic marker after receiving treatment for HCC have shown variable results. The determination of AFP levels is not usually included in most prognostic and therapeutic algorithms for HCC, except for those patients eligible for liver transplantation. Some studies, such as the one conducted by Dominguez et al. [44], have indicated that patients with elevated AFP levels have a higher risk of recurrence after liver transplantation. Thus, levels >1000 ng/mL have been considered exclusion criteria for liver transplantation in large hospital centers [18]. In line with the previous study, the research led by Baj et al. establishes that elevated AFP levels prior to surgical resection are associated with a worse prognosis after surgery and a higher risk of recurrence [44,45]. In the meta-analysis carried out by He et al., AFP levels were assessed in patients diagnosed with HCC after receiving treatment. Despite a significant disparity in treatment response, AFP levels emerge as a promising non-invasive prognostic marker in this type of tumor, particularly in those who underwent curative-intent treatment. Consequently, the decline in AFP levels after liver transplantation or surgical resection was associated with an increase in overall survival, extended progression-free survival, and recurrence-free survival in patients with HCC [46]. However, other studies, such as the one conducted by Schlosser et al., do not indicate a strong correlation between AFP levels and the prognosis of the disease. They suggest the implementation of a combination of different biomarkers to improve the treatment of these patients [47]. Moreover, according to the clinical trial by Zhu et al., an improvement in overall survival has been demonstrated in patients with advanced disease who had AFP levels exceeding 400 ng/mL and showed no response to sorafenib. These patients were treated with ramucirumab as a second-line drug [48].

In the conducted study, the etiology of HCC was the most important variable in predicting mortality at the time of diagnosis, followed by the BCLC classification, which proved superior to TNM. The BCLC classification, widely used in our context, includes the degree of hepatic functional reserve through the Child–Pugh scale, in addition to the tumor’s own characteristics as in the TNM. Alcohol consumption was the etiological factor associated with a worse prognosis. This cause was not only isolated but also worsened the prognosis when associated with other causes of HCC, such as chronic hepatitis C infection. This fact may be because alcohol is one of the most associated etiological factors with the development of liver cirrhosis. Also, closely monitoring these patients is challenging, making early detection of HCC more complex [49]. However, elevated levels of AFP were not associated with lower survival in these patients. Therefore, despite being one of the most commonly used serological biomarkers to date, its isolated determination was not related to the prognosis of these tumors. AFP has different isoforms according to the composition of sugars during the enzymatic glycosylation process [34,50]. Three different isoforms of AFP with varying affinities for lectin binding, such as Lens culinaris agglutinin (LCA), have been identified. These isoforms are known as AFP-L1, AFP-L2, and AFP-L3 [32,50]. AFP-L3 binds more strongly to LCA and is the predominant isoform in patients with HCC, especially in those with small tumors (<3 cm) [32,51]. Its determination could be very useful in the early diagnosis and prognosis of HCC. It has already been employed in several studies, including one by Ido et al. It involves an automated immunoassay by electrophoresis, demonstrating that it is a biomarker capable of increasing sensitivity and specificity, especially in patients with serum AFP values below 20 ng/mL [52,53].

In line with the study conducted by Schlosser et al. [47], according to research by Cagnin et al., the combination of different variables such as gender, age, AFP-L3 levels, AFP, and des-carboxyprothrombin (DCP) grouped under the term GALAD score could be useful in detecting these tumors at earlier stages, proving to be a promising prognostic tool [54]. The conduct of further studies to enhance our understanding of prognostic factors in HCC at the time of diagnosis would be advisable. The development and implementation of new prognostic biomarkers could prove beneficial in clinical practice, aiming to improve survival and enable personalized management for these patients.

## 4. Materials and Methods

### 4.1. Study Design and Population

A multicenter retrospective cohort study was conducted at the Virgen de la Luz Hospital in Cuenca and the University Hospital of Guadalajara. All patients diagnosed with HCC from 2008 to 2022 were included in the study, totaling 208 cases. Inclusion criteria encompassed patients aged 18 and above diagnosed with HCC through either histological examination or imaging techniques. Exclusion criteria applied to patients with a previous diagnosis from another facility without knowledge of prognostic variables at the initial diagnosis. The study received approval from the Ethics Committee of the University Hospital of Guadalajara, and obtaining informed consent was deemed unnecessary.

### 4.2. Study Data

The study included variables generally associated with the progression of HCC. Demographic variables encompassed gender and age at the time of HCC diagnosis. Age was defined as the difference between the diagnosis date and the date of birth. The censoring date for each patient in our study corresponded to the date of death for deceased patients and the date of the last medical visit for those who remained alive. Variables related to toxic habits acquired by patients were analyzed. Among these, alcohol consumption was recorded, with harmful consumption defined as >30 g/day in males and >20 g/day in females [55]; smoking status was categorized as being a smoker or former smoker compared to those who had never smoked. Variables related to metabolic syndrome included type 2 diabetes mellitus, defined according to the medical history by fasting glucose ≥ 126 mg/dL and/or glucose tolerance test > 200 mg/dL 2 h after glucose overload [56]; the presence of dyslipidemia was determined based on medical history and/or the use of lipid-lowering medications [57]; body mass index (BMI), calculated using the formula weight (kg)/height^2^ (m^2^), was used to define obesity (BMI ≥ 30 kg/m^2^) [58].

The patient’s health status was defined according to the ECOG general status scale [59]. The presence of cirrhosis was defined based on clinical and radiological criteria [60]; the diagnosis of HCC was achieved through invasive or radiological methods in cirrhotic livers with typical behavior, distinguishing between patients included in HCC screening programs with semi-annual ultrasound and those not undergoing close surveillance. Different etiologies related to the development of these tumors were considered, such as alcohol, HCV, HBV, MASLD, hemochromatosis, autoimmune hepatitis, primary biliary cirrhosis, Wilson’s disease, porphyrias, aflatoxins, and alpha-1 antitrypsin deficiency. According to the degree of functional hepatic reserve, some of the most representative variables were examined, such as the Child–Pugh classification, MELD. The presence of clinically relevant portal hypertension was defined by a hepatic venous pressure gradient greater than 10 mmHg, the presence of esophagogastric varices, or the presence of ascites [61]. Regarding the tumor’s own characteristics, the number of space-occupying lesions (SOL), the size of the largest SOL in cm, and the presence of portal thrombosis, pathological lymph nodes, or metastases at the time of diagnosis were recorded. Two of the most widely used prognostic and therapeutic classifications, namely BCLC and TNM, were also collected [62].

The analytical values included bilirubin (mg/dL), albumin (g/dL), INR, Na (mEq/L), lymphocytes (cells/mm^3^), neutrophils (cells/mm^3^), platelets (cells/mm^3^), CRP (mg/L), AFP (ng/mL), creatinine (mg/dL), AST (U/L), and alanine aminotransferase (ALT) (U/L).

### 4.3. Development Model

For the statistical analysis, the variables were collected in an anonymized database. The analysis focused on prognostic factors for HCC at the time of diagnosis using machine learning (ML) methods.

XGB is a predictive algorithm characterized by its utilization of boosting techniques within a supervised learning framework. Boosting involves the sequential generation of multiple “weak” prediction models, where each subsequent model leverages the results of the preceding one to create a more “robust” model with enhanced predictive power and result stability. The optimization algorithm used, specifically gradient descent, contributes to refining the model’s strength. Throughout the training process, the parameters of each weak model are iteratively adjusted in an attempt to minimize an objective function [63,64]. When presented with a dataset set (x_i_, y_i_), the XGB algorithm was formulated as:

(1) $$\hat{y_{i}}=\sum_{p=1}^{P}t_{p}\left(x_{i}\right)$$

In this context, x_i_ denotes the input featuring m time variables, y_i_ signifies the output, $\hat{y_{i}}$ denotes the predicted output, t_p_ represents a tree characterized by leaf weight w_p_ and structure up, where i ranges from 1 to n, and P corresponds to the total number of trees.

Equation (2) introduces the regularized objective function for the proposed method, showcasing a deviation from traditional ensemble methods. In this case, the suggested approach leverages a second-order Taylor expansion to approximate the target function of XGB, ultimately elevating the precision of prediction [63,64].

(2) $$R=\sum_{i}r\left(\hat{y_{i}},y_{i}\right)+\sum_{p}\Phi \left(t_{p}\right)$$

(3) $$\Phi \left(t_{p}\right)=\lambda f_{p}+\frac{1}{2}\gamma {\left\|\omega_{p}\right\|}^{2}$$

To control the method’s complexity and prevent overfitting, a regulatory term, denoted by weights, serves as a monitoring mechanism. Described in Equation (3), *f_p_* signifies the tree trimming utilized for overfitting control, representing the number of leaves in the tree; λ denotes the learning rate; and *w* is the vector of scores assigned to the leaves. The function R() assesses the disparity between the target output y_i_ and the predicted output $\hat{y_{i}}$. The function Φ punishes the complexity of the system. The parameter γ is employed to regulate the complexity weight of the system [63,64]. In pursuit of enhanced performance, the objective of this work is to minimize Equation (2).

**Figure 5:**
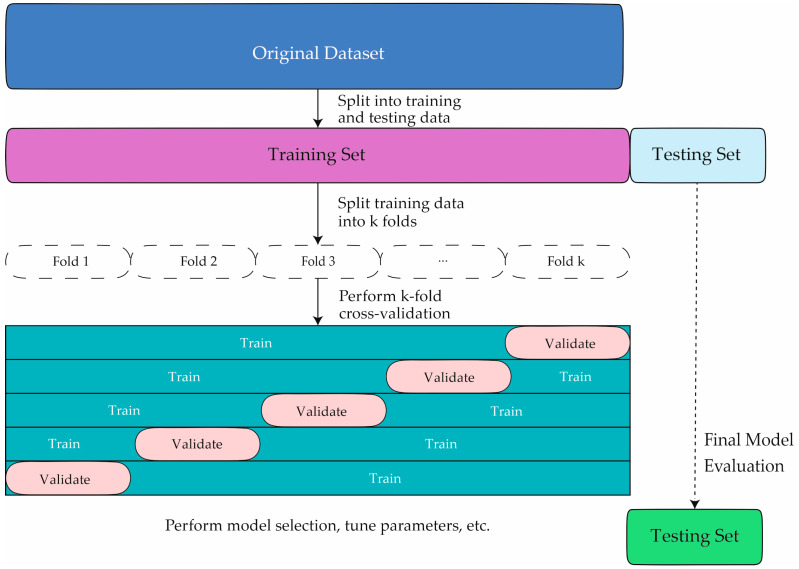
The figure shows the processes followed in this study for the development of machine learning models.

In the machine learning system learning process, it is necessary to control overfitting. In our case, the k-fold cross-validation technique was employed for this purpose. As can be seen in Figure 5, each iteration involves the random classification of 70% of patients for training and 30% for validation. Specifically, patient data are not shared between the training and validation subsets to prevent the algorithm from being validated with data from the same patients used in the training phase. In this study, bootstrapped resampling techniques have been applied. In this approach, a subset of the available labeled data is randomly sampled with replacement, creating a new training set. By generating multiple bootstrapped training sets, a machine learning model can be trained on each set, thus creating multiple models. These models are then combined to make predictions. The bootstrapping process introduces randomness and diversity in the training sets, allowing the models to capture different aspects of the data and reduce overfitting [65]. We have also used the data augmentation technique. This technique is widely used in machine learning to increase the amount of training data. This helps to improve model generalization and avoid overfitting [66].

To enhance the performance of the machine learning algorithms, various hyperparameters of each method were fine-tuned during the training phase. Bayesian techniques were employed in this study to determine hyperparameter values. Bayesian optimization is a type of optimization algorithm based on sequential models, utilizing the results of previous interactions to refine parameter tests in subsequent experiments. This approach reduces the number of times a model needs to be tested for validation, focusing only on hyperparameters expected to yield superior validation scores. The optimization method significantly improved the performance of the developed models.

The proposed XGB algorithm was chosen to develop the predictive model due to its scalability, high execution speed, and support for parallel computing, which are its main advantages over other machine learning methods. XGB also allows second-order regularization, aiding in preventing a common machine learning issue, overfitting, by enhancing model generalization. Therefore, the XGB algorithm exhibits high accuracy and proves to be more efficient than other algorithms in data analysis through machine learning [21,67]. Machine Learning Toolbox and MATLAB Statistics (The MathWorks, Natick, MA, USA; MATLAB 2023) were used to design the models. The proposed XGB method was compared with other ML algorithms such as SVM [68], DT [69], GNB [70], KNN [71], and BLDA [72].

The most prominent hyperparameters of the implemented systems are as follows. For the SVM method, a Gaussian kernel function is chosen with the following parameters: C = 1, sigma = 0.5, numerical tolerance = 0.001, and iteration limit = 100. For the DT system, the base parameter estimator is adjusted: Tree, maximum number of splits = 20, learning rate = 0.1, and number of learners = 40. GNB algorithm: usekernel: False, fL = 0 and Adjust = 0. As for the BLDA algorithm, the Bayesian kernel has been selected. For the KNN method, the distance metric is Euclidean, and it uses 20 neighbors. Finally, for the XGB system, the hyperparameters eta = 0.2, minimum chil weight = 1, gamma = 0.3, alpha = 0.5, maximum depth = 9, lambda = 0.3, col sample by tree = 0.5, and maximum delta step = 5 have been adjusted.

The preference for the proposed XGB over other alternative machine learning algorithms is based on its notable advantages, positioning it as a superior choice in terms of robustness, accuracy, and versatility [73].

Compared to SVM, XGB showcases a distinctive ability to handle intricate and high-dimensional datasets while maintaining computational efficiency. Its ensemble approach inherently introduces diversity, reducing the risk of overfitting and producing more generalized and predictive models, particularly in situations with heightened problem complexity.

In contrast to GNB, XGB excels at effectively managing irrelevant or noisy features. The integration of multiple independent decision trees allows the model to dis-miss less informative variables, significantly improving robustness and predictive efficacy.

Unlike KNN, which may be sensitive to noisy data, XGB demonstrates inherent resilience to dataset noise and variability. By constructing models based on multiple trees, the impact of outliers or errors is mitigated, ensuring greater reliability in decision making.

To sum up, the preference for XGB is substantiated by its ability to generate robust and accurate predictive models, particularly in complex environments and large datasets. Its resistance to overfitting, capability to handle irrelevant features, and versatility relative to other algorithms make it a favored choice, ensuring more dependable results and enhancing the model’s generalization capabilities.

## 5. Conclusions

In conclusion, the currently widely used AFP isoform lacks utility as a prognostic factor for mortality at the diagnosis of HCC. Other variables, such as the presence of alcohol as a cause of HCC or the BCLC score, are more useful and provide more information.

The proposed XGB method has successfully developed a valuable diagnostic tool for predicting mortality in HCC patients. Through this model, the primary predictive factors influencing the objective of this study have been identified. The XGB algorithm yielded the best results for the analyzed metrics, exhibiting no overfitting or excessive tuning. This system has demonstrated a high model generalization capacity, rendering it a valuable tool in daily clinical practice.

Further studies involving additional isoforms of AFP, alongside exploration of other biomarkers, are necessary to more accurately predict mortality in these patients. This approach will also facilitate the optimization of patient management and aid in determining the most effective treatments for this population. This will also facilitate optimizing their management and determining the best treatments for these patients. Utilizing ML algorithms, particularly XGB, can be highly beneficial in assessing the utility of these new parameters.

## Figures and Tables

**Table 1:** Set of results of the mean values and standard deviations of accuracy, AUC, precision and recall obtain of the different machine learning models and XGB method in the study. SVM: support vector machine, BLDA: Bayesian linear discriminant analysis, DT: decision tree, GNB: Gaussian naïve Bayes, KNN: K-nearest neighbors, XGB: eXtreme gradient boosting, AUC: area under the curve.

	Methods					
	SVM	BLDA	DT	GNB	KNN	XGB
Accuracy	86.72 ± 0.84	82.03 ± 0.93	84.65 ± 0.81	80.52 ± 0.95	89.34 ± 0.57	95.68 ± 0.36
AUC	0.87 ± 0.02	0.82 ± 0.02	0.85 ± 0.02	0.81 ± 0.02	0.89 ± 0.01	0.95 ± 0.01
Precision	86.10 ± 0.82	81.45 ± 0.89	84.05 ± 0.79	79.94 ± 0.93	88.71 ± 0.55	94.97 ± 0.33
Recall	86.82 ± 0.79	82.13 ± 0.87	84.75 ± 0.77	80.61 ± 0.91	89.45 ± 0.54	95.70 ± 0.32

**Table 2:** Set of results of the mean values and standard deviations of F_1_ score, kappa, DYI and MCC obtain of the different machine learning models and XGB method in the study. SVM: support vector machine, BLDA: Bayesian linear discriminant analysis, DT: decision tree, GNB: Gaussian naïve Bayes, KNN: K-nearest neighbors, XGB: eXtreme gradient boosting, MCC: Matthews correlation coefficient, DYI: degenerated Youden index.

	Methods					
	SVM	BLDA	DT	GNB	KNN	XGB
F_1_ score	86.46 ± 0.81	81.78 ± 0.89	84.40 ± 0.77	80.28 ± 0.91	89.08 ± 0.54	95.06 ± 0.33
Kappa	77.21 ± 0.57	73.03 ± 0.61	75.36 ± 0.52	71.68 ± 0.65	79.54 ± 0.42	84.74 ± 0.29
DYI	86.72 ± 0.83	82.03 ± 0.90	84.65 ± 0.80	80.52 ± 0.94	89.34 ± 0.56	95.18 ± 0.34
MCC	76.95 ± 0.55	72.79 ± 0.60	75.11 ± 0.51	71.45 ± 0.63	79.28 ± 0.41	84.46 ± 0.28

## Data Availability

The datasets used and/or analyzed during the present study are available from the corresponding author on reasonable request.
